# Trimodal Waveguide Demonstration and Its Implementation as a High Order Mode Interferometer for Sensing Application

**DOI:** 10.3390/s19122821

**Published:** 2019-06-24

**Authors:** Jhonattan C. Ramirez, Lucas H. Gabrielli, Laura M. Lechuga, Hugo E. Hernandez-Figueroa

**Affiliations:** 1 Department of Electronic Engineering, School of Engineering, Federal University of Minas Gerais (UFMG), Belo Horizonte 31270-901, MG, Brazil; 2 School of Electrical and Computer Engineering (FEEC), University of Campinas (UNICAMP), Campinas 13083-852, SP, Brazil; lucashg@fee.unicamp.br (L.H.G.); hugo@decom.fee.unicamp.br (H.E.H.-F.); 3 Nanobiosensors and Bioanalytical Group, Catalan Institute of Nanoscience and Nanotechnology (ICN2), CSIC, BISTCIBER-BBN, 08193 Bellaterra (Barcelona), Spain; llechuga@cin2.es

**Keywords:** integrated optics, modal interferometer, sensor, polymer waveguide, direct laser writer fabrication

## Abstract

This work implements and demonstrates an interferometric transducer based on a trimodal optical waveguide concept. The readout signal is generated from the interference between the fundamental and second-order modes propagating on a straight polymer waveguide. Intuitively, the higher the mode order, the larger the fraction of power (evanescent field) propagating outside the waveguide core, hence the higher the sensitivity that can be achieved when interfering against the strongly confined fundamental mode. The device is fabricated using the polymer SU-8 over a SiO_2_ substrate and shows a free spectral range of 20.2 nm and signal visibility of 5.7 dB, reaching a sensitivity to temperature variations of 0.0586 dB/°C. The results indicate that the proposed interferometer is a promising candidate for highly sensitive, compact and low-cost photonic transducer for implementation in different types of sensing applications, among these, point-of-care.

## 1. Introduction

Interferometers represent one of the most used and affordable devices for telecommunications [1,2,3,4], sensing [5,6,7,8,9], and other applications in fundamental physics [10,11]. The progress of the field of photonic sensors, with the implementation of devices such as the ring resonators or different types of interferometers, has allowed the fabrication of miniaturized sensing devices exhibiting high sensitivity (with limit of detection between $10^{-6}$ and $10^{-8}$ refractive index units), fast response and, label-free and real-time monitoring capabilities [12] in applications related with biomedical sensing.

However, despite these advances, the mass production of this type of device as a low-cost, disposable sensor is unlikely due to high production costs. As an alternative, the use of polymers [13] to fabricate optical waveguide sensors represents a viable and attractive alternative [14,15,16].

Additional advantages by using polymers are the fabrication flexibility and the low power consumption [17,18]. These polymeric photonic sensors can be at the core of the innovative technology platform called photonic lab-on-a-chip (PhLOC) [7,19,20,21,22], aimed towards solving existing limitations in achieving low cost mass production devices for point-of-care diagnosis [23,24,25]. Even though the aim of the PhLOC is to integrate all the required elements—photonic and non-photonic—in a stand-alone system, there has been no demonstration that this configuration can achieve the claimed high sensitivity in a label-free evaluation [7,26]. In this scenario, the most standard photonic configuration is based on the light-analyte interaction thought, the evanescent field (EF). In conventional waveguides, the EF carries around 2% of the total confined energy at the highest. Thus, centimeter-range interaction lengths are required to provide a reliable signal. An additional issue is that inhomogeneity in the waveguide dimensions of less than 1% can generate variations in the behavior of light propagating in the photonic device, affecting the resulting signal [27,28].

In order to address those challenges, we proposed in a previous work [29] a high sensitive polymer based interferometric sensor, which was the starting point for works developed later [30] and bases its operating principle on the interaction between the fundamental and the second-order mode of an optical waveguide acting, respectively, as reference and sensor mode. In this work we experimentally demonstrate the trimodal interferometric sensor based on polymer technology. The device is fabricated on SU-8 in a single exposition step, which indicates the possibility of very low mass-production costs. Nonetheless, it is still capable of reaching high sensitivity, comparable to more complex designs or more expensive material stacks [31,32,33,34].

## 2. Results and Discussion

### 2.1. Trimodal Interferometer Concept and Simulations

**Figure 1:**
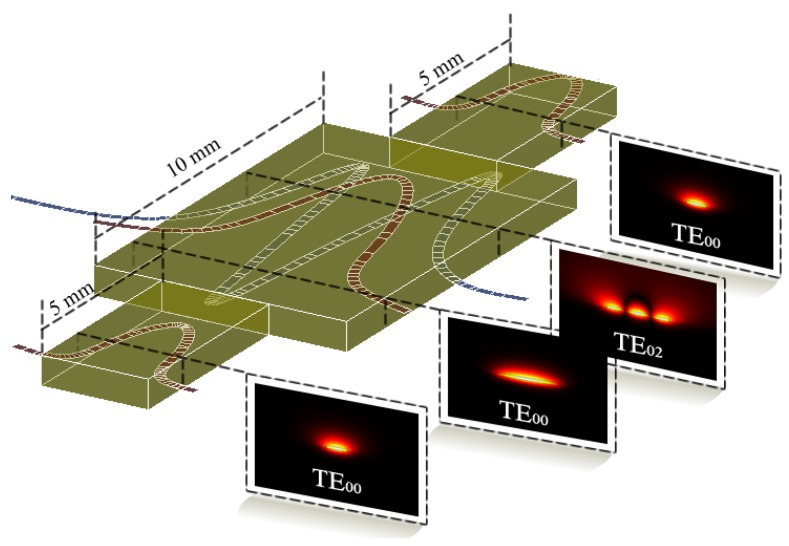
Scheme of the Trimodal waveguide sensor showing the expected light modes in each section.

As illustrated in Figure 1, the sensor is composed of two single-mode waveguides used as input and output for the trimodal section. The coupling to the trimodal section exploit the symmetry of the modes so that only the fundamental (TE_00_) and second-order (TE_02_) modes of the trimodal section are efficiently excited, leaving out the first-order one (TE_01_).

In this sense the fundamental mode is highly confined in the waveguide core, being relatively insensitive to external perturbations. On the other hand, the second-order displays an extensive evanescent tail, making it very susceptible to changes in the cladding region (sensing area) in comparison to the fundamental mode [29]. Therefore, the coherent sum of both contributions in the single-mode output section described by:

(1) $$\begin{array}{c} d=\sum_{n=0}^{N}c_{n}e^{-j\beta_{n}L}\frac{{\;\int \int \;}_{S}{\vec{e}}_{n}\times \vec{H}\;\cdot \;d\hat{s}}{{\;\int \int \;}_{S}\vec{E}\times \vec{H}\;\cdot \;d\hat{s}}, \end{array}$$

where ${\left|d\right|}^{2}$ is the power fraction at the output of the interferometric device. It represents an interferometric signal that can be directly related to changes in the sensing area.

The visibility of the interferometric signal is directly affected by the balance in excitation of both modes of interest in the trimodal section. The different mode profiles that are excited in each device section are also presented in Figure 1.

Ideally, when both modes are excited with the same efficiency, their destructive interference would exactly cancel each other, maximizing the interference fringe at the output. However, noise and scattering processes limit this ideal case; moreover, perfectly balancing the excitation of the modes is a challenging task in the proposed material platform: it is much easier to transfer power to the fundamental mode than to the second-order one due to its similarities to the fundamental mode in the single-mode section. In order to minimize this difference, the geometry of each section must be carefully chosen.

For a 600 nm thick waveguide core layer, the widths of the single-mode and trimodal sections were varied between 2 μm and 6 μm, and between 10 μm and 12 μm, respectively, while the length of the latter was fixed to 10 mm.

**Figure 2:**
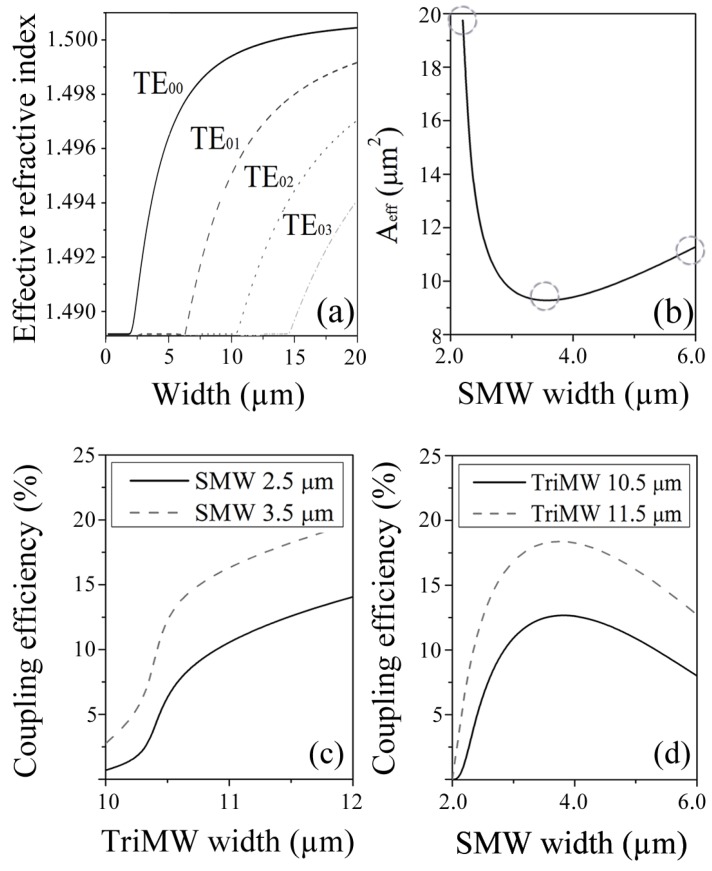
(**a**) Cutoff analysis for 600 nm height waveguide. (**b**) Effective area analysis as function of the width variation in a single mode section waveguide at the single channel modal interferometer with 600 nm height, working at 1550 nm wavelength. Analysis of the percentage of energy coupled in the second order mode as function of the width variation in a (**c**) single mode section and in the (**d**) multimode section, at the same wavelength.

The fabricated device will be highly affected by polarization changes, because the dimensions of the designed component as shown in Figure 2a are very close to the cutoff region for the second order mode at TE polarization. In case of variations in the polarization, the desired modes will not be excited and therefore we would not get the desired interference at the output.

Figure 2b shows the variation of the effective mode area in the single-mode section as function of the waveguide width. Working at 1550 nm wavelength, was reached the smallest effective area [35] at 9.26μm^2^ when the device has a width of 3.5 μm.

A smaller transverse dimension leads to weaker confinement, therefore with a longer evanescent tail, increasing the effective area. However, although a wider core results in a more confined mode, the mode area grows as a result of the larger core itself. The impact of the widths of the single-mode and trimodal sections in the coupling efficiency to the second-order mode are displayed in Figure 2c,d, respectively. Because of the fields distribution in the second-order mode, coupling to the single-mode waveguide benefits from a reduction in the effective modal area of the latter, as a maximum is reached for the single-mode width of 3.5 μm. Regarding the width of the trimodal section, the larger it is, the more efficient the coupling. However, this dimension must be kept below the cut-off of higher order modes, which would contribute to additional loss in visibility of the interferometric signal.

### 2.2. Direct Laser Writer Fabrication Procedure

Analyzing the thermal conductivity (TC) of SU-8 (0.30 W/mK), which is similar to the TC of teflon (0.25 W/mK) and much lower than that of the silicon (130 W/mK), it was possible to guarantee a low variability of the refractive index in the core for the fabricated structures, due to controlled temperature changes as suggested [36]. Other parameters such as viscoelasticity, Young’s modulus, Poisson ratio, thermal hysteresis, liquid–solid-phase transition and losses as function of temperature changes, are of great interest for this work; according to results presented in previously published papers by our group [37], and other contributions by other research groups [36,38], we can say that the SU-8 is an excellent material to be used in MEMS and Integrated Photonics (IP), because of its great thermal and structural stability, even being exposed to extreme conditions, such as high temperatures, i.e., around 150 °C for long periods of time.

Devices with different widths in a single- and multimode sections were fabricated in order to verify the previous coupling analysis through the comparison of their interferometric signals visibility and resulting sensitivity.

The implemented material for the modeling and fabrication of the described devices was SU-8, which is a high refractive index and highly transparent photo-resist that must be polymerized at 365 nm wavelength. Taking into account the initial conditions of the material chosen to compose the core of our devices, it was necessary to develop the manufacturing process using Direct Laser Writer (DLW), at 405 nm wavelength [37], in two stages: in the first stage we dissolved the SU-8 2100 to achieve the desired thickness, i.e., 600 nm. The resultant polymer was mixed with the photoinitiator H-nu 470, generating the displacement of the absorption peak of the material from UV to the visible spectrum, allowing the fabrication of the proposed devices using the DLW equipment. The SU-8 solution was stirred about 48 h after receiving 0.1 wt.% of the photoinitiator H-nu 470, 2.5 wt.% of OPPI photoacid generator and 0.1 wt.% of cationic cure accelerator AN-910E [37].

The second stage involves the preparation of the sample, which followed the usual SU-8 processing: after cleaning in piranha bath, the oxidized Si substrate with a 2 μm SiO_2_ layer was dehydrated on a 200 °C hot plate for 20 min, spin coated with SU-8 and pre-baked at 95 °C for 1 min. The SU-8 was mixed with H-nu 470 (Spectra Group) to allow exposure in a Heidelberg DWL 66FS system at 405 nm wavelength and 75.6 mJ/cm^2^ dose [37]. Afterwards, the sample was subjected to a post-exposure bake at 95 °C for 1 min. The fabricated structures were then developed in SU-8 developer solution by immersion and stirring for 30 s, then hard-baked for 5 min at 150 °C on a hot plate. A top cladding based on PMMA (polymethyl methacrylate), was deposited on the fabricated interferometric devices: 950 PMMA A7, 4% in anisole was spin-coated and hard-baked for 5 min on a 150 °C hot plate.

Thanks to the modifications in absorption, induced by the implemented photoinitiator, it was possible to obtain devices with a transmission rate, greater than 95% in the area of interest, and thermal resistance at temperatures below 400 °C, before and after the pre- and post-exposure bake, as can be seen in more detail in [37,39].

Multiple devices have been fabricated using the described procedure developed by our group, demonstrating repeatability and consistency in the results and devices obtained, as can be seen in [13].

**Figure 3:**
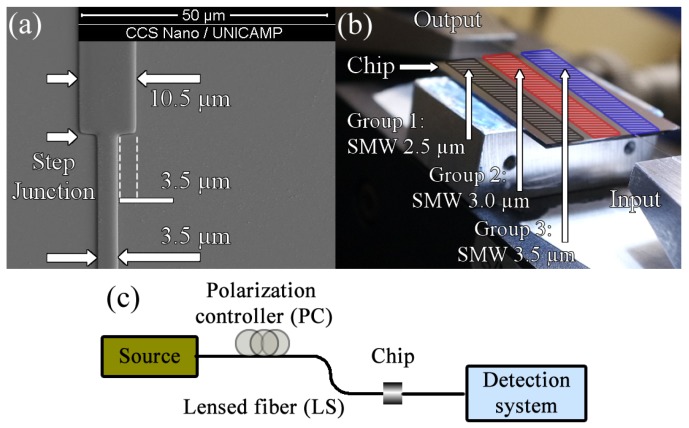
Fabrication and characterization system of the trimodal interferometer device. (**a**) The SEM image of a trimodal device, highlighting the single- and multimode area and the step junction section, respectively. (**b**) The experimental setup for the trimodal interferometers. (**c**) Design of the experimental setup to characterize the manufactured interferometric devices.

We fabricated a chip with eighteen components divided into three blocks, each block with one single mode waveguide as reference and five devices with a trimodal section as shown in the scanning electron microscope (SEM) picture in Figure 3a.

As previously said, the cross section of the manufactured devices has 600 nm in height and variable width between 10 μm and 12 μm with a 0.5 μm pitch. In Figure 3a, it can be observed that the region called step junction, i.e., where the single mode waveguide and the multimode section are connected, is well-defined by the lithography process, therefore the excitation of the desired modes is guaranteed.

As illustrated in Figure 3b, each group is differentiated by the width of the single mode section of the components, i.e., 2.5 μm, 3 μm and 3.5 μm, respectively. The measured fabrication error was 22.8 nm approximately.

### 2.3. Optical Characterization

In the experiments, a Keysigth 81 636B and a 81 980A tunable laser C band at 8 dBm with Electric Field Polarized (EFP) is coupled to the input waveguide through a polarization rotator and a lensed single-mode fiber aligned with the help of high precision piezo-electric stages, as shown in Figure 3b,c. Similarly, the output of the signal is collected via a lensed fiber and delivered to a calibrated power meter. The measured propagation losses in a single mode (SM) waveguide were 0.57 dB/cm at 1550 nm wavelength.

The interferometric devices on chip have a multimode section of 10 mm in length and two single-mode sections of 5 mm in length each, for a total length of the components of 20 mm.

**Figure 4:**
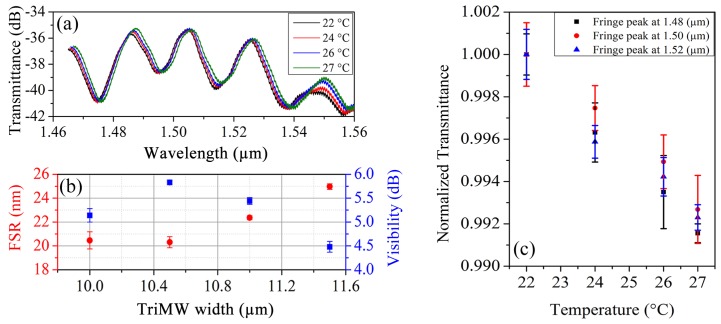
Sensitivity measurement in trimodal devices. (**a**) Measured interferometric signal resulting from the modal interaction within a trimodal interferometer with 3.5 μm width in the single mode section and 10.5 μm width in the multimodal region. (**b**) FSR and visibility analysis for a trimodal interferometric device with 600 nm height and cross-section with single mode section width of 3.5 μm and variable multimode section width between 10 μm and 12 μm, with a 0.5 μm pitch. (**c**) Normalized transmittance as function of temperature variation for fringe peaks at 1485 nm, 1505 nm and 1525 nm.

A sample interferometric signal obtained from the device with single-mode section width of 3.5 μm and trimodal section width of 10.5 μm is shown in Figure 4a. In this configuration the coupling efficiency to the fundamental and second-order modes are 77% and 13%, respectively, which results in a signal visibility of about 5.8 dB and a free spectral range (FSR) of 20.3 nm.

The sensitivity of an interferometric sensor will depend strongly on the visibility of the signal and the FSR reached by the photonic component, by guaranteeing a small FSR and high visibility, a highly sensitive device will be obtained.

The FSR will strongly depend on the group index difference $\Delta n_{g}$ between the modes that interact within the multimodal section, i.e., the fundamental mode and the second-order mode, and the total length of interaction *L*, because both parameters are inversely proportional to the FSR. Due to the extensive evanescent wave of the second order mode, it is possible to observe that by varying the wavelength, we obtain a well-defined interferometric signal, as illustrated in Figure 4a. In the same image, the intensity response of an interferometer at different temperatures can be appreciated, where the device preserves its FSR and visibility despite the induced temperature variations, however, it is possible to appreciate variation in the final transmittance and wavelength shift because of the mentioned changes.

Variations in the width of the trimodal section can be controlled to improve visibility or free spectral range depending on whether the detection method is based on power measurement, wavelength tuning, or a combination of both, as shown in Figure 4b.

As presented in Figure 4c, it is possible to observe more clearly the behaviour of the normalized transmittance for the fringe peaks at 1485 nm, 1505 nm and 1525 nm when the temperature is varied. The obtained results are very similar, however, the fringe peak at 1485 nm shows a greater variation of the signal and lower error, when compared with the other two mentioned cases. A fourth fringe peak at 1550 nm was not taking into account, because the visibility obtained by the fabricated interferometer is seriously affect due to the dimensions proximity to the cutoff region, as presented in Figure 2a. In this sense, between 1540 nm and 1560 nm the interferometer intensity response is not appropriate.

Surface roughness and fabrication error, seriously affect the homogeneity of the manufactured devices and consequently the obtained results by the designed structures. In this sense, as said previously, we have 22.8 nm of fabrication error measured in the manufactured trimodal devices, in addition, and according to what was reported in [37], the surface roughness in fabricated devices by Direct Laser Writer technique developed by our group is 0.44nm±0.05nm. Taking into account these manufacturing imperfections, we measured ten devices of each of the dimensions in the trimodal section described previously, obtaining the results presented in Table 1.

**Figure 5:**
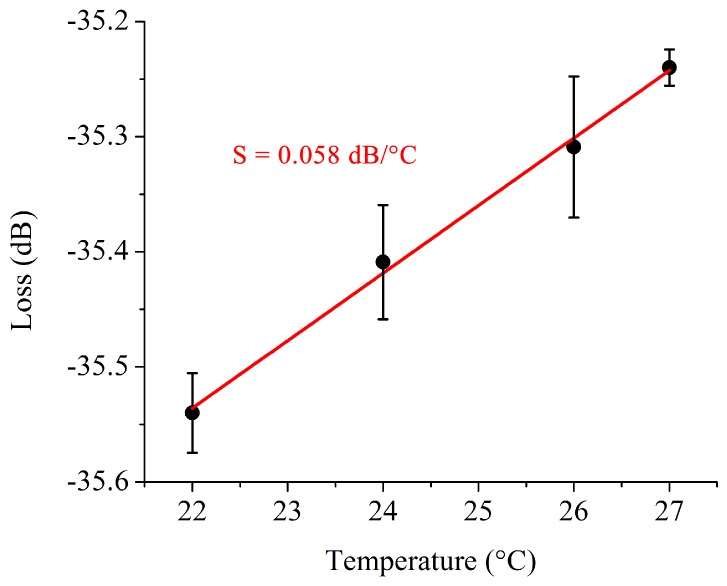
Fringe power measured as function of temperature variation at 1485 nm wavelength peak.

The measured insertion loss at 1550 nm wavelength, including both coupling was around 20 dB. Excluding the I/O coupling losses, in total, we have losses around 15 dB and 21 dB in our trimodal waveguide because of the losses in the step junction section. The results obtained are displayed in Figure 5.

In order to evaluate the performance of the trimodal interferometric devices, due to the strong interaction of the second order mode with the surrounding area, an interferometric device with 3.5 μm width in the single mode sections, 10.5 μm in the multimode section and 600 nm height for both regions was used. This configuration was selected by the strong confinement of the fundamental mode in the multimode section, extensive evanescent field of the second order mode and high coupling coefficient in the step junction at the input and output of the multimodal section.

The high order mode interferometer was tested as a temperature sensing device to probe its sensitivity. By varying the temperature from 22 °C up to 27 °C, it was possible to observe changes in the resulting interferometric signal as shown in Figure 5. By adjusting a line to the measured transmission data at 1486 nm the obtained temperature sensitivity was 0.059 dB/°C. This value is twice as high as those found in the state-of-the-art for compact and low-loss fiber optical sensors [40].

Sensitivity studies were carried out varying the refractive index in the sensor area, for the fabricated device and for simulated optimized device with the same structural composition.

**Figure 6:**
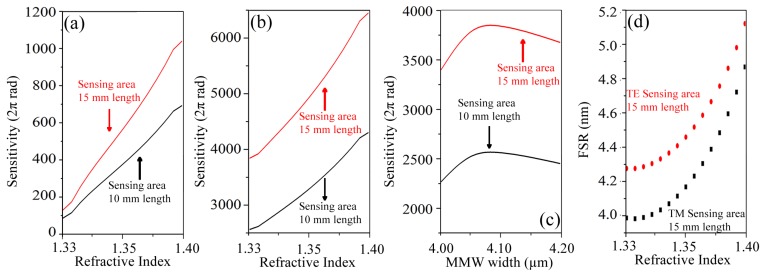
Bulk sensitivities for 10 mm and 15 mm length in the sensing area. (**a**) Simulated sensitivity for fabricated devices, as function of refractive index variation on the sensing area, for TE propagation mode. (**b**,**c**) Simulated sensitivity for optimized trimodal component, as function of refractive index variation on the sensing area, and variation of the width dimensions in the trimodal region, respectively. (**d**) Free Spectral Range for the optimized trimodal component.

The refractive index on sensing area of the simulated fabricated device was varied between 1.33 and 1.40, for 10 mm and 15 mm length in that region, reaching results that vary between 100 (2π rad) and 1100 (2π rad), respectively, as shown in Figure 6a. It is very important to note that the obtained values are comparable with reported simulated sensitivities by devices such as Surface Plasmon Resonance (SPR) biosensors, photonic crystals biosensors, among others [7].

On the other hand, we optimized our devices to obtain the better result as possible, taking into account 10 mm and 15 mm length in the sensing area as well, Figure 6b,c. The achieved sensitivities vary between 2500 (2π rad) and 6500 (2π rad) for the same previously presented refractive index variation, i.e., between 1.33 and 1.40. In this sense, we can say that our polymeric based trimodal interferometer has the potential to reach sensitivities, similar and even higher to those reported in interferometric devices based on silicon technology, although the implemented polymeric materials have a lower refractive index than the silicon one.

Finally, was analyzed numerically the behavior of the FSR for the optimized trimodal device, and it is possible to appreciate a 4 nm FSR, when the refractive index on sensing area is 1.33, as can be seen in Figure 6d, which is an unusual result for polymer technology based devices.

Besides high sensitivity, another advantage of the demonstrated interferometric sensor is the possibility of implementing tens or hundreds of devices in a compact chip, allowing multiplexed detection in parallel for, as an example, complex disease diagnosis in biosensing applications, as required for future PhLOC systems.

## 3. Conclusions

In this work, the design, fabrication and optical characterization of a compact high order mode interferometric sensor based on modal interaction into trimodal waveguide was presented and demonstrated. The sensor is fabricated on a low cost platform, leveraging polymer technology to produce disposable devices suitable for mass production and point-of-care applications. Parameters such as the coupling efficiency, free spectral range and signal visibility were investigated numerically and experimentally, showing that the widths of each device section can be controlled to tune each parameter for any preferred detection method. Finally, the device was evaluated as a temperature sensor and showed a sensitivity of 0.058 dB/°C at room temperature. Such sensitivity indicates that the trimodal interferometer is a promising candidate for label-free detection in biosensing applications.

## Figures and Tables

**Table 1:** FSR and visibility detailed data.

TriMW Width	FSR	Visibility
10.0μm	20.46nm±0.7220nm	5.14nm±0.142dB
10.5μm	20.31nm±0.4635nm	5.83nm±0.0349dB
11.0μm	22.36nm±0.1654nm	5.44nm±0.0721dB
11.5μm	24.97nm±0.2327nm	4.48nm±0.1134dB
